# Research Progress and New Ideas on the Theory and Methodology of Water Quality Criteria for the Protection of Aquatic Organisms

**DOI:** 10.3390/toxics11070557

**Published:** 2023-06-25

**Authors:** Chenglian Feng, Wenjie Huang, Yu Qiao, Daqing Liu, Huixian Li

**Affiliations:** 1 State Key Laboratory of Environmental Criteria and Risk Assessment, Chinese Research Academy of Environmental Sciences, Beijing 100012, China; fengcl@craes.org.cn (C.F.);; 2 College of Water Science, Beijing Normal University, Beijing 100875, China

**Keywords:** water quality criteria (WQC), species sensitivity distribution (SSD), assessment factor, aquatic organisms, model prediction, emerging pollutants

## Abstract

Water quality criteria (WQC) for the protection of aquatic organisms mainly focus on the maximum threshold values of the pollutants that do not have harmful effects on aquatic organisms. The WQC value is the result obtained based on scientific experiments in the laboratory and data fitting extrapolation and is the limit of the threshold value of pollutants or other harmful factors in the water environment. Until now, many studies have been carried out on WQC for the protection of aquatic organisms internationally, and several countries have also issued their own relevant technical guidelines. Thus, the WQC method for the protection of aquatic organisms has been basically formed, with species sensitivity distribution (SSD) as the main method and the assessment factor (AF) as the auxiliary method. In addition, in terms of the case studies on WQC, many scholars have conducted relevant case studies on various pollutants. At the national level, several countries have also released WQC values for typical pollutants. This study systematically discusses the general situation, theoretical methodology and research progress of WQC for the protection of aquatic organisms, and deeply analyzes the key scientific issues that need to be considered in the research of WQC. Furthermore, combined with the specific characteristics of the emerging pollutants, some new ideas and directions for future WQC research for the protection of aquatic organisms are also proposed.

## 1. Introduction

Water quality criteria (WQC) refers to the maximum concentration or level at which pollutants or environmental factors in water environments do not have harmful effects on human health or water ecosystems [1,2,3]. WQC is the scientific basis for water quality standards and plays an important role in environmental protection and management. According to the different protective receptors, WQC can be roughly divided into the protection of aquatic organisms and the protection of human health. The most important factors affecting the WQC for aquatic organisms are the toxic effects of pollutants, biota differences, water quality parameter and the extrapolation methods of the WQC. There are differences in biota in different countries/regions, which leads to different WQC values even for the same pollutant [4,5,6]. WQC studies in different countries are carried out on the basis of their own regional environment. The environmental behavior and bioavailability of pollutants in different regions may be different; thus, WQC values are also obviously regional [7,8]. Some countries have issued corresponding guidelines for the protection of aquatic life WQC and technical documents for several pollutants.

Currently, the two mainstream WQC research systems internationally are based on those of the United States and the European Union (EU). The United States puts forward the toxicity percentage ranking method, which is a two-value criteria system [9]. It pointed out that the toxicity data used to derive WQC should cover at least three phyla and eight families of organisms and provide adequate protection for most biological species (more than 95%). In recent years, WQC methods have been improved, and the species sensitivity distribution (SSD) method has been gradually adopted to extrapolate the WQC. Some countries or organizations represented by the EU adopted the SSD method as the extrapolation method of WQC [10]. With the deepening of research, data screening and model optimization have been developed and improved [11]. In terms of the expressions of WQC value, they can be expressed numerically and narratively according to different indicator categories and extrapolation methods of WQC. Most of the numerical values are expressed as the concentrations of pollutants in water environments. For those criteria that cannot give specific numerical indicators, such as color and turbidity, narrative criteria are often used [12,13].

Various relevant factors are comprehensively considered in the derivation process of WQC, and the determination of criteria values is influenced by many water environmental factors, such as water hardness, temperature, pH and dissolved organic matter, and so on [14,15,16,17]. Especially in the WQC studies of heavy metals, many countries will calibrate the toxicity values with water environment parameters. At present, the common correction methods for specific regional WQC in different countries and regions include single water quality parameter correction (i.e., hardness correction or pH correction), dual water quality parameter correction, and multiple water quality parameter correction [18,19,20]. In the 1980s, when it was fully realized that the toxicity of metals is determined by their interactions with other components in the water, the US EPA began to derive the WQC of metals based on a function of hardness [21,22]. This is a big improvement over the previous methods, but the method does not take into account the effects of other factors such as temperature, pH, dissolved organic carbon, sulfides, and alkalinity on metal toxicity. The WQC of pentachlorophenol in the United States takes pH as an important consideration, and the WQC is finally expressed as a function of pH. The most representative case of two-parameter correction is Canada. When formulating long-term WQC for manganese, Canada has conducted hardness correction for fish and invertebrate toxicity data and pH correction for plant toxicity data [23]. The most representative method for multiple water quality parameter correction is the biological ligand model (BLM) [24,25]. At present, Cu-BLM is the most mature BLM applied to heavy metals in freshwater environments. In addition, BLM models for heavy metals such as silver, cadmium, zinc, nickel, cobalt, and lead are also being established and developed. The criteria for copper published in the United States are derived using the BLM [26]. Meanwhile, some studies have also used multiple linear regression methods to study the toxicity and WQC of heavy metals [17,27,28].

Based on the mentioned above, the main purpose of the present study is to systematically summarize and integrate the current technical methods of WQC, deeply analyze the key issues and technical systems of WQC research methods and summarize the current case studies of WQC. Finally, some new ideas of the derivation method of WQC for emerging pollutants are also explored and prospected.

## 2. Interpretation of WQC Guidelines for the Protection of Aquatic Organisms in Different Countries

The pioneering study on WQC began in the early 20th century. The United States was the earliest country to conduct WQC research. The development process of water quality standards is presented in the form of a series of WQC papers, reports, and monographs. Additionally, the United States finally issued its national water quality guideline for the protection of aquatic organisms in 1985. In addition, the European Union, the Netherlands, Canada, Australia and New Zealand, Japan, China, and other countries have also formulated their own relevant technical guidelines for WQC and established their own WQC and ecological risk assessment research systems. With the deepening of WQC research, many countries have revised, improved, and updated their guidelines in recent years (Table 1).

For example, the technical guidance of the United States was issued in 1985 [9], and the main method used is the toxicity percentage ranking method. The main core of this method is to arrange the average toxicity values of the species from small to large, to select the four most sensitive genera, and to use a series of formulas to calculate the final criteria values. The criteria values obtained by use of this method include the criteria maximum concentration (CMC) and criteria continuous concentration (CCC). CMC considers the acute toxic effect of pollutants on aquatic animals, which is equal to half of the final acute value; CCC considers the chronic toxic effect of pollutants on aquatic animals, which is equal to the minimum of final chronic value, final plant value and final residual value.

The European Union promulgated the Water Framework Directive (WFD) in 2000, which played an important role in developing and promoting the setting of water environmental quality standards. The WFD is a legal framework designed to protect freshwater and marine ecosystems from the adverse effects of pollutants and to protect human health. The European Union (EU) uses environmental risk assessment technology to derive the predicted no-effect concentration (PNEC) of pollutants as water quality protective objectives for environmental management and issued the “*Technical Guidance Document for Risk Assessment*” (TGD) in 2003 [8]. In 2004, at the request of the European Commission for the Environment, the Fraunhofer Institute (FHI), based on TGD Guidelines, prepared a technical guidance manual on the establishment of WQC for priority pollutants of the WFD. In 2007, under the framework of the EU’s WFD, the expert group on environmental quality criteria conducted the compiling work of environmental quality criteria in the field of water environments. The event is led and organized by the United Kingdom and the joint research center with support from a working group of experts from EU member states. In 2011, the EU issued the technical guidelines for the derivation of environmental quality criteria, which were updated in 2018 [29]. This is also a programmatic document that has been used to guide WQC research.

In addition to the two mainstream countries of the United States and the European Union, some other countries and organizations have also conducted relevant research on WQC. For example, the Organization for Economic Cooperation and Development (OECD) issued the *Guidance Document for Aquatic Effects Assessment* in 1995 to evaluate the hazardous effect of pollutants on water environments [30]. In 1999, the Canadian Council of Ministers of the Environment (CCME) first published “*A protocol for the derivation of water quality guidelines for the protection of aquatic life”* [31], which was revised and improved in 2007 [32]. The Australian and New Zealand Environmental Protection Council and Agriculture and Resource Management Council (ANZECC/ARMCANZ) issued “*Australian and New Zealand guidelines for fresh and marine water quality”* in 2000 [33], which was revised and updated in 2018 [34]. The National Institute of Public Health and the Environment (RIVM) of the Netherlands published *the Guidance document on deriving environmental risk limits* in 2001 [35], which was revised and improved in 2007 [36]. The Ministry of Environment of Japan published *Environmental Quality Standards for Water Pollution* in 1971 [37], which was revised and improved in 2021 [38].

The research on WQC in China started relatively late, dating back to the 1980s. The initial research was only the collection and collation of relative references and data and integrated the research methods of WQC in different countries and organizations. Subsequently, on the basis of a large number of theoretical explorations and WQC case studies, some monographs and books related to WQC were published one after another [4,5,39]. The technical guide document at the national level is “*Technical guideline for deriving WQC for freshwater organisms*”, which was first released by the Ministry of Ecology and Environment in 2017 [40]. It is also the first technical guideline in the field of environmental criteria in China. In 2022, some details of the guidelines were improved and revised for the first time [3]. The methodology of China’s WQC guideline is to fully absorb and refer to the latest international WQC research methods, and recommend the SSD method as the extrapolation method of WQC. Meanwhile, a supporting national standardization software named “EEC-SSD” was also developed for WQC derivation [41], which is also an important innovation and highlight in China’s research on WQC.

## 3. Key Points of Theoretical Methodology of WQC for the Protection of Aquatic Organisms

There are many key steps involved in the extrapolation of WQC for the protection of aquatic organisms. In general, the factors affecting WQC mainly include the reliability of basic data, standardization of toxicity data and the scientificity of statistical analysis methods. All these elements play a very important role in the study of WQC.

Firstly, in terms of species selection, the selection of regional native species is crucial for WQC. Many studies have shown that, for the same pollutant, biota differences will lead to the ultimate difference in the WQC value of the pollutant, which further highlights the importance of site-specific or native species [4,42,43]. Therefore, the desired species need to be selected according to regional characteristics and differences in biota. Different countries have defined the quantity requirements for species selection. For example, the US EPA, the European Union, Canada, Australia and New Zealand have requirements on the number of species selected, including at least three trophic levels of fish, invertebrates and plants, and at least five species before subsequent data fitting and WQC extrapolation [11]. After the completion of a species screening process, the acquisition and screening of species toxicity data are also important parts. The quality of the obtained data should also be evaluated according to the reliability and relevance of the data. Klimisch et al. [44] divided toxicity data into four levels according to reliability, correlation and appropriateness and generally screened the data of level 1 and level 2 for WQC research. For example, the European Union evaluates the quality of data according to the principles of reliability and relevance [10], while the UK, the Netherlands, Canada, Australia, New Zealand and China all adopted similar data screening principles [5,11,32,33,36].

**Figure 1:**
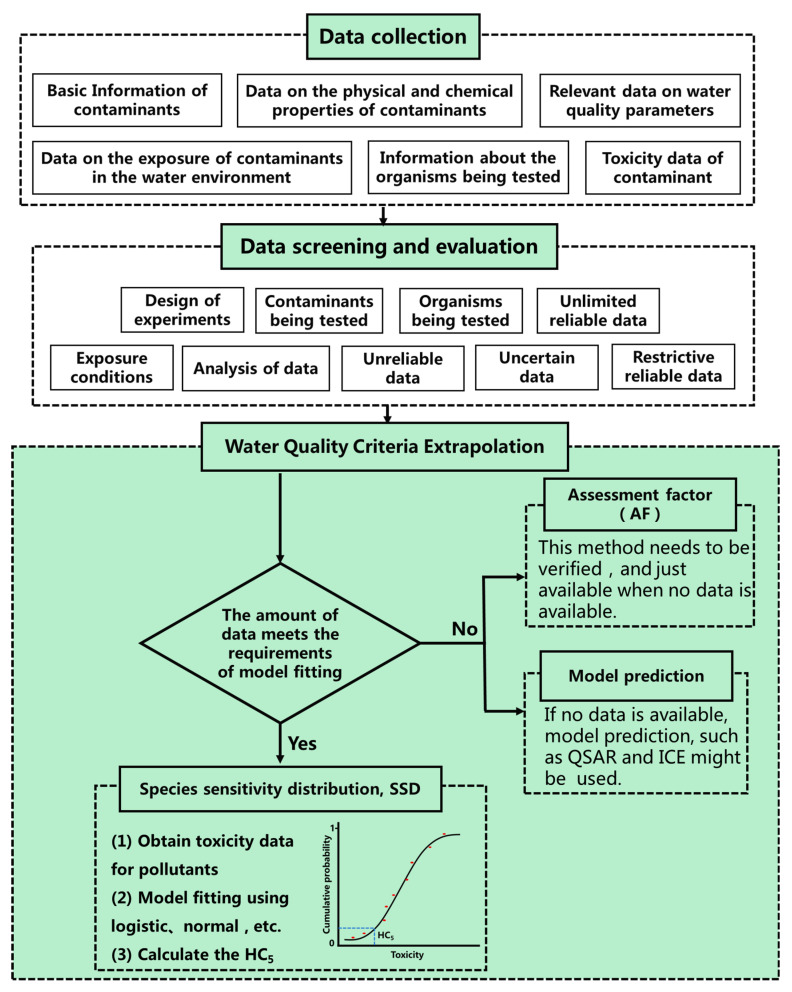
The main process and method of water quality criteria derivation.

Secondly, in terms of WQC derivation methods, the derivation system based on the SSD method and supplemented by the assessment factor (AF) method has been basically formed in the world at present. The main steps of WQC derivation mainly include data collection, data screening and evaluation, and WQC derivation (Figure 1). The basic idea of the AF method is to divide the most sensitive toxicity data of the pollutant by the assessment factor to obtain the WQC value of the pollutant. The AF method requires less basic data, and the calculation method is simple. The disadvantage is that this method is empirical and depends on the toxicity value of sensitive organisms, with high uncertainty. In addition, the AF method does not consider the relationship between species and the biological enrichment effect of pollutants. Therefore, it is only used when the data are difficult to obtain or for comparative verification. In contrast, the advantage of the SSD method is that it makes full use of the toxicity data available for all species and assumes that a limited number of species are randomly sampled from the ecosystem and can be representative of the entire ecosystem. It uses all the toxicity data of known pollutants to fit the sensitivity distribution curve of species and then extrapolates the WQC value. The criteria value is the concentration corresponding to a specified percentage point on the curve, usually expressed as HC_5_, which is the concentration at which 5% of species are at risk [3,45,46]. In terms of the scope of use, when the toxicity data of pollutants are relatively sufficient, the SSD method is used to fit the model and extrapolate the criteria, which will statistically reduce the uncertainty. It can be seen that the SSD method has become the international trend of WQC methods and will also be the main method of WQC research in the future. The guidelines for WQC issued by most countries are mainly based on the SSD method. Only the guidelines issued by Canada in 1999 [31] only used the AF method for WQC derivation. However, after the revision in 2007 [32], the SSD method was added. In addition, the principle of giving priority to the SSD followed by the AF method was also proposed.

The SSD method is an international mainstream method used to establish ecological environment criteria. Model selection is the core and key of this method. When building SSD models, the commonly used derivation methods according to the amount of toxicity data include the normal distribution model, logistic distribution model, etc., and the mainstream trend is to use the best-fit curve to derive the final WQC value [47,48,49]. Some mathematical statistics software, such as Origin, Matlab, Sigmaplot and other software commonly used in mathematical statistics are mostly used. The fitting results of different models and different statistical software will have certain differences, which may lead to differences in model selection and calculation results of different scholars [50]. In order to solve this problem, some countries have developed or stipulated their own national environmental criteria calculation software. For example, the Netherlands recommended using EcoToX software. Burrlioz software was recommended in Australia and New Zealand [34]. The United States has developed an SSD toolbox for fitting SSD using Fortran language programming [51]. Based on the experience of other countries and combined with empirical data, China developed and first released national ecological environment criteria calculation software–species sensitivity distribution method, EEC-SSD in 2022 [41], which provided standardized technical support for the setting of national ecological and environmental criteria. The release of standardized software at the national level mentioned above provides a guarantee for the fine management of the environmental criteria. With the continuous deepening of model research methods, some relatively mature models have gradually been recommended by WQC researchers and recommended as mainstream derivation models, such as the Burr III model [34].

Thirdly, in terms of the description and value of the WQC for specific pollutants, the double-value criteria system is mainly used internationally at present. The United States, Canada, China, and other countries adopt double-value criteria. Double-value criteria generally refer to long-term criteria and short-term criteria (or chronic criteria and acute criteria). Long-term criteria (chronic criteria) are designed to protect aquatic life (all species and their life stages) from the negative effects caused by the indefinite long-term effects of pollutants. In contrast, short-term criteria are designed to protect aquatic organisms from serious negative effects (such as death) caused by short-term effects. Some countries and organizations also use single-value criteria such as the European Union [8]. There are also countries where the criteria value is neither double-value nor single-value but is classified according to the different protection levels. For example, in Australia and New Zealand, the WQC were divided into four level criteria, i.e., 99%, 95%, 90% and 80% of the criteria values according to the scope of the protected objects (Table 1). In addition, there are also several differences in the description or naming of WQC. For example, in the United States, they were named CMC and CCC. In China, the WQC were divided into short-term WQC for aquatic organisms (SWQC) and long-term WQC for aquatic organisms (LWQC). In Canada, they were used as water quality guidelines (WQG) for the protection of aquatic life, which were divided into long-term concentration and short-term concentration. The European Union directly uses PNEC to represent its WQC. Additionally, in Australia and New Zealand, they were termed trigger values (TVs) in the 2000 Guidelines [33] and named water quality guideline values (GVs) in the updated 2018 Guidelines [34].

## 4. Research Progress of Case Studies of WQC for Environmental Pollutants

### 4.1. Bibliometric Analysis of WQC Research

**Figure 2:**
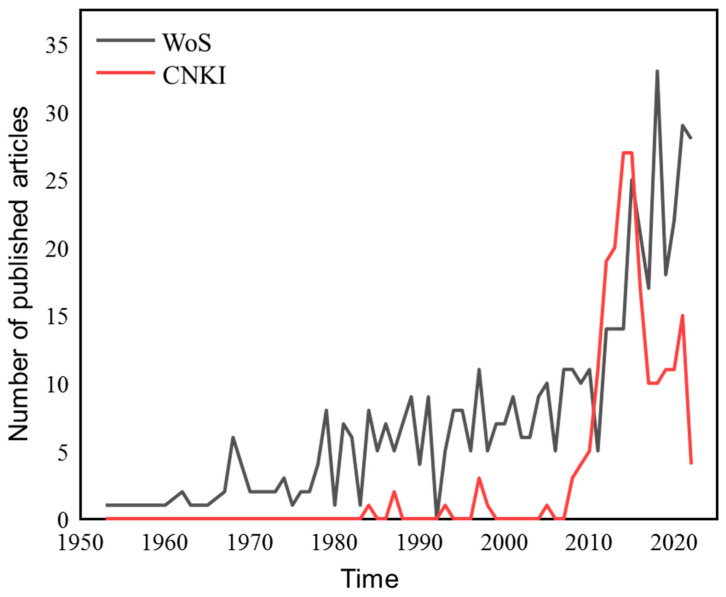
Schematic diagram of the trend of the annual average number of articles published by WoS and CNKI on water quality criteria (Note: WoS is Web of Science; CNKI is China National Knowledge Infrastructure).

In recent years, great progress has been made in international research focusing on WQC. Many scholars have carried out a large number of WQC case studies of typical pollutants and published a series of research papers, monographs, etc. These studies not only summarized and explored the theoretical methodology of WQC for the protection of aquatic organisms but also provided case studies on WQC, including conventional pollutants, some physical and chemical parameters of water bodies, and new emerging pollutants [4,5,11,16,39,45,52]. Bibliometrics uses statistical methods to conduct quantitative analysis of scientific papers, which can describe the research status and emerging trends in this field and explore future research hotspots and directions [53]. CiteSpace is a citation visualization analysis software based on scientific metrology data and information visualization technology for analyzing potential information in the literature [54,55]. This software can not only analyze the co-citation of literature and mining clustering information, but also analyze the cooperation information of authors, institutions, and countries/regions. Based on this, bibliometrics was used to statistically analyze the previously published papers. The retrieval deadline was September 2022. With the theme of “WQC”, “water quality guidelines” and “water quality standards”, relevant articles were retrieved on Web of Science (WoS) and China National Knowledge Infrastructure (CNKI), respectively. The results showed that a total of 501 and 203 relevant articles (704 articles in total) were retrieved on WoS and CNKI, respectively (Figure 2). The first article on WQC was published in 1953 and was retrieved in WoS [56]. Since then, the annual average number of documents issued has been less than 10. Since 2010, the number of articles related to WQC has increased rapidly, reaching a peak in 2015. The research on WQC in CNKI started late. The first article related to WQC was published in 1984 [57], and the number of articles published gradually increased from 2010 but showed a downward trend in recent years.

**Figure 3:**
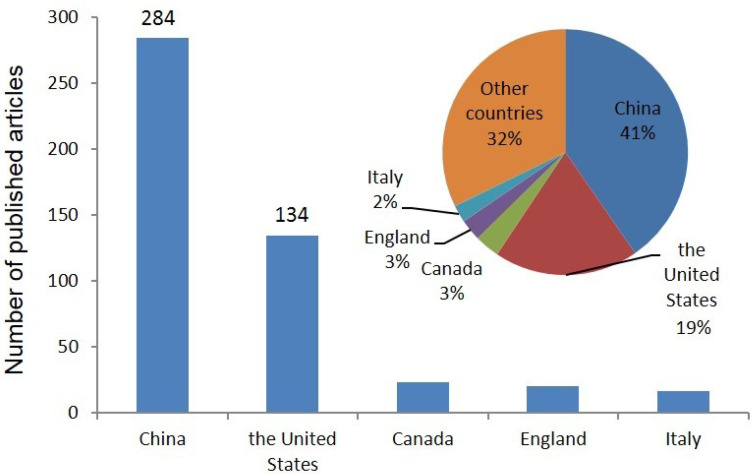
Number of articles published on water quality criteria in different countries.

At the same time, the countries and organizations studying WQC were screened, and it was found that China and the United States were significantly ahead of other countries in terms of the number of publications on WQC, with 284 and 134 publications (41% and 19% of the total, respectively). In China, the Chinese research academy of environmental sciences published the most articles, and its secondary institution, the state key laboratory of environmental criteria and risk assessment, contributed more than 118 articles. The top five countries in terms of the number of articles issued were China, the United States, Canada, the United Kingdom, and Italy. Additionally, the number of WQC articles issued by these five countries accounted for 60% of all the published articles (Figure 3).

### 4.2. WQC Values of Toxic Substances Published at the National Level

In terms of the WQC values of toxic substances published at the national level, some countries have also published WQC for the protection of aquatic organisms for some toxic substances at the national level. For example, since the release of the guidelines for WQC in the United States, a number of WQC for toxic substances have been published, and the WQC are updated almost every 2–3 years in combination with the latest research. EPA’s compilation of nationally recommended WQC is presented as a summary table containing recommended WQC for the protection of aquatic life and human health in surface waters. These criteria are published pursuant to the Clean Water Act and provide guidance for states and tribes to use to establish water quality standards and ultimately provide a basis for controlling discharges or releases of pollutants. At present, the latest WQC published by the US EPA contains 186 indicators [21,22], including 61 indicators to protect aquatic life (including 31 indicators of organic matter, 21 indicators of inorganic matter and 9 other indicators, such as pH, temperature, dissolved oxygen, etc.) and 125 indicators to protect human health (Table 2).

In addition to the United States, Australia and New Zealand [58], Canada [59], Japan [60] and China [61,62,63] have also published WQC for some toxic substances at the national level. Based on the water quality guideline, Australia and New Zealand also published the default guideline values (DGVs) of the pollutants in fresh water and seawater, which were updated in 2023. DGVs were derived using toxicity data for at least three species from at least three taxonomic groups. The listed criteria indicators included a total of 159 freshwater WQC. The WQC value published by Canada contains 194 toxic substance items. In Japan, there are only three indicators in the criteria for the protection of aquatic life. That is, total zinc, nonylphenol and straight-chain alkylbenzene sulfonate, and their criteria values are determined according to the type of water and the type of organism. Up to now, China has only published WQC for toxic substances, namely cadmium, ammonia nitrogen and phenol, and all of them were published in 2020 [61,62,63].

## 5. Exploration of New Theories and Methods of WQC Research

### 5.1. Exploration of WQC Research Methods Based on Model Prediction

The acquisition of toxicity data is the key issue of WQC research. Different types of pollutants have different influencing factors in the study of WQC. For example, the toxic effect of organic matter on organisms is relatively complex, so the endpoint of the toxic effect of organic pollutants on organisms should be clarified in the WQC study, and then the criteria are deduced. The toxic effects of heavy metals are greatly influenced by environmental factors. Another problem encountered in WQC studies is the lack of data on the toxicity of the selected pollutants. The toxicity data could not meet the requirements of fitting the SSD curve, nor could they meet the requirements of the AF method. However, when environmental management is in urgent need, it is necessary to predict the toxicity data of pollutants by means of model prediction. Model prediction includes the following levels. First, how to use laboratory experiments to predict actual toxicity effects in the field? The Biotic Ligand Models (BLM) approach can be used. The BLM is a mechanistic approach that greatly improves our ability to generate site-specific ambient water quality criteria for metals in the natural environment [26]. Water environmental factors (such as hardness, organic matter content, pH, etc.) have a great influence on metal toxicity. The BLM allows metal–organism interactions to be taken into account and given site-specific information on actual water chemistry to evaluate the dissolved metal concentration associated with a critical level of metal accumulation that is toxic to an organism. It serves as a powerful tool for predicting metal toxicity because it accounts for the concurrent influences of several environmental factors that alter site-specific metal bioavailability in an organism. In contrast to full BLMs, which require the input of field data on upwards of 10 parameters, simplified BLMs integrate data on dissolved metal concentrations, pH, DOC, and calcium concentrations to predict the amount of metal available for uptake by organisms on a site-specific basis, such as Cu, Zn, Ni, Pb (BIO-MET, M-BAT and PNEC-pro) and Mn (M-BAT). At present, most toxicity data are based on laboratory toxicity tests, which mainly consider the total concentration of metal and do not reflect the concept of bioavailability. In fact, the toxicity of metals is closely related to the form of metals, on the one hand, and also closely related to water environmental factors such as organic matter content, hardness, pH value and so on. That is, the concentration of the effective state can better reflect the actual toxicity of the metal. Therefore, the impact of water environmental factors on their bioavailability should be taken into consideration when studying the criteria of such pollutants [4,64,65]. For example, in the WQC for the protection of aquatic organisms published in the United States [26], the copper criteria adopted the method of BLM method, which fully considered the impact of water environmental conditions on acute copper toxicity and its criteria. The European Union (EU) has launched a European Union Water Framework Directive. WQC for copper and other metals that have been developed for use within this regulatory framework are typically based on site-specific effect levels that are evaluated by means of chronic BLMs. In addition to the above-mentioned models, there are also some Multiple Linear Regression (MLR) models [17,66,67], normalized SSD models, etc., which some scholars are trying to adopt for the study of WQC. Second, how to use the toxicity of known species to predict the toxicity of unknown species? Some scholars proposed the use of Interspecies Correlation Estimation (ICE) models and verified the applicability of this model through case studies [68,69]. ICE was first developed by the US EPA, with the surrogate species toxicity data to predict the toxicity data of other unknown species [70]. The basic principle is that there is a linear relationship between the toxicity of the replacement species and the predicted species so that the toxicity of other species can be predicted based on the known toxicity data of the replacement species. At present, it is only used to predict acute toxicity. Thus, the purpose of supplementing the toxicity data of unknown species and then deriving the WQC can be realized. Third, how can the chemical structure of a pollutant be used to predict its toxic effects? Quantitative structural activity correlation (QSAR) models can be used for experiments, and it has been shown that metal toxicity and WQC can also be derived using QSAR models [16,71].

### 5.2. New Ideas on WQC Research for Emerging Pollutants

Emerging pollutants refer to the pollutants produced in production, construction or other activities, which are caused by human activities, that clearly exist but have not been regulated by laws, regulations and standards and harm the living and ecological environment. With the continuous detection of emerging pollutants in the environment, they are gradually receiving widespread attention [72,73,74]. The first characteristic of emerging pollutants is that they are “new”. There are many kinds of emerging pollutants. At present, there are more than 20 categories of emerging pollutants of global concern, and each category contains dozens or hundreds of chemical substances. This mainly includes persistent organic pollutants (POPs), endocrine-disrupting chemicals (EDCs), antibiotics and microplastic, etc. The second characteristic is “high environmental risk”, which is mainly reflected in the severity of the hazard, the hidden risk, the persistence of the environment, the extensive source, and the complexity of the management. Therefore, it is very important to study the WQC and risk assessment of emerging pollutants.

Compared with conventional pollutants, emerging pollutants have the following differences. Taking EDC as an example, first, the dose–response relationship of conventional pollutants generally follows the principle of “low dose, low toxicity”, and its toxicity value has a certain threshold. While for EDCs, it is also toxic at low doses, with the characteristics of “low dose, high toxicity”. In addition, EDCs may exhibit certain biological effects, including delayed and multigenerational effects, and may exhibit non-monotonic dose–response relationships [75,76]. Secondly, in terms of the selection of sensitive species, the requirements for the derivation of conventional pollutant WQC include fish, amphibians, invertebrates, aquatic plants, algae, etc. However, aquatic plants, algae and some lower invertebrates do not have endocrine systems, which is not suitable for the derivation of WQC for EDCs. Therefore, toxicity data of aquatic plants, algae and lower invertebrates should not be considered when deriving WQC for EDCs [43,55]. Moreover, the toxic effects of conventional pollutants are generally growth and death. While, for EDCs, their toxic effects are complex and changeable, including delayed effect, non-monotonic effect, and more generation effect, which has a great deal of uncertainty. It is necessary to identify its sensitive toxic effect endpoint and select the most sensitive toxic effect endpoint suitable for such substances, such as development, reproduction, and other sensitive effects, to derive the WQC.

Furthermore, in terms of WQC values, when deriving WQC for conventional pollutants, double criteria values, such as short-term criteria values and long-term criteria values, are generally formulated. Nonetheless, EDCs in a short time exposure generally will not cause serious toxicity on aquatic organisms, while, under trace concentrations, will have irreversible toxicity effects on aquatic organisms. The acute-to-chronic ratio of EDCs is generally large; the largest may reach 10,000 or 100,000 times. Therefore, it is recommended to use single-value criteria when deriving WQC for EDCs, and only the values obtained from chronic toxicity are used to derive the long-term criteria value. In addition, it is also important to establish an effective correlation between toxic effects and endpoints [77]. Many effects, from the molecular level to the individual level, are used to evaluate the endocrine mechanism of different groups, but the relationship between these effects and the group-level endpoint is often unclear. If new methods are applied, such as adverse outcome pathways (AOPs) and population modeling, the relationship between EDCs at the level of low-level biological tissues and adverse endpoints at the population level can be better understood. An AOP framework should be built by fully utilizing the existing toxicity data of EDCs based on in vivo and in vitro to completely and deeply understand their toxic mechanisms. Through molecular initiation events (MIEs), key events (KEs) and adverse outcomes (AOs), AOP can organically arrange the existing pollutants’ toxic effect mechanisms and toxicity endpoints and provide a theoretical basis for WQC research and predict the toxicity of new pollutants in the near future [74]. At present, most of the toxicity data of chemical pollutants are based on single-species toxicity tests at the individual or organizational level. However, the safety threshold derived from individual-level toxicity testing cannot guarantee population safety. The ecological threshold based on population modeling is of great significance for the protection of aquatic biodiversity and the structure and function of the entire aquatic ecosystem [78]. In Japan, population modeling approaches that have been applied to the threshold of some high-priority chemicals such as nonylphenol, polychlorinated biphenyls, and tributyltin included the derivation of the predicted-no-effect concentration (PNEC) for medaka population-level impact based on population growth rate, as well as the lowest observed-effect concentration (LOEC), the no-observed-effect concentration (NOEC), and the maximum-acceptable-toxic concentration (MATC) [79].

In addition, a non-monotonic dose–effect relationship may appear in both in vitro and short-term in vivo studies of the EDCs, but it might not be able to widely predict the toxicity endpoint in long-term in vivo studies. In the absence of the toxicity threshold of EDCs, probabilistic methods should be used to predict the threshold, which is also a new idea for the future study of WQC of emerging pollutants.

## Figures and Tables

**Table 1:** Technical guidelines for water quality criteria for the protection of aquatic life in different countries/organizations.

Country /Organization	Technical Guidelines	Release Time	Description
the United States	Guidelines for deriving numerical national WQC for the protection of aquatic organisms and their uses	1985 (first release)	CMC, CCC
European Union (EU)	Technical guidance document on risk assessment-partⅡ	2003 (first release)	PNEC
	Common implementation strategy for the water framework directive (2000/60/EC) guidance document No. 27 technical guidance for deriving environmental quality standards	2018 (latest edition)	
OECD	Guidance Document for Aquatic Effects Assessment	1995 (first release)	HC_5_
Canada	A protocol for the derivation of water quality guidelines for the protection of aquatic life	1999 (first release)	WQG
	A protocol for the derivation of water quality guidelines for the protection of aquatic life	2007 (latest edition)	
Australia and New Zealand	Australian and New Zealand guidelines for fresh and marine water quality	2000 (first release)	TV GV
	Revised method for deriving Australian and New Zealand water quality guideline values for toxicants	2018 (latest edition)	
The Netherlands	Guidance document on deriving environmental risk limits	2001 (first release)	ERL
	Guidance for the derivation of environmental risk limits within the framework of ‘International and national environmental quality standards for substances in the Netherlands’ (INS) Revision 2007	2007 (latest edition)	
Japan	Environmental Quality Standards for Water Pollution	1971 (first release)	EQS
	Environmental Quality Standards for Water Pollution	2021 (latest edition)	
China	Technical guideline for deriving WQC for the protection of freshwater aquatic organisms	2017 (first release)	WQC
	Technical guideline for deriving WQC for freshwater organisms	2022 (latest edition)	

Note: CMC is criteria continuous concentration; CCC is criteria maximum concentration; PNEC is predicted no-effect concentration; HC_5_ is 5 percent hazardous concentration; WQG is water quality guideline; TV is trigger value; GV is guideline value; ERL is environmental risk limit; EQS is environmental quality standard; WQC is water quality criteria.

**Table 2:** Summary of WQC for aquatic life in the United States.

Indicator Categories	The Specific Name	Number
Organic substances	4,4′-DDT, Acrolein, Aldrin, alpha-Endosulfan, Atrazine, beta-Endosulfan, Carbaryl, Chlordane, Chlorpyrifos, Cyanide, Demeton, Diazinon, Dieldrin, Endrin, gamma-BHC (Lindane), Guthion, Heptachlor Epoxide, Heptachlor, Malathion, Methoxychlor, Methyl Tertiary-Butyl Ether (MTBE), Mirex, Nonylphenol, Oil and Grease, Parathion, Pentachlorophenol, Perfluorooctane Sulfonate (PFOS), Perfluorooctanoic Acid (PFOA), Polychlorinated Biphenyls (PCBs), Toxaphene, Tributyltin	31
Inorganic substances	Alkalinity, Aluminum, Ammonia, Arsenic, Boron, Cadmium, Chloride, Chlorine, Chromium (III), Chromium (VI), Copper, Hardness, Iron, Lead, Mercury, Nickel, Phosphorus Elemental, Selenium, Silver, Sulfide-Hydrogen Sulfide, Zinc	21
Other indicators	Aesthetic Qualities; Color;Gases, Total Dissolved; Nutrients; Oxygen, Dissolved Freshwater; pH; Solids Suspended and Turbidity; Tainting Substances; Temperature	9
Total		61

## Data Availability

Not applicable.
